# Research Participant Interest in Alzheimer’s Disease Biomarker Disclosure

**DOI:** 10.1093/geroni/igaa057.3251

**Published:** 2020-12-16

**Authors:** Claire Erickson, Nathaniel Chin, Carey Gleason, Sterling Johnson, Lindsay Clark

**Affiliations:** 1 University of Wisconsin-Madison, Madison, Wisconsin, United States; 2 University of Wisconsin School of Medicine and Public Health, Madison, Wisconsin, United States

## Abstract

Researchers can characterize the pathophysiological progression of Alzheimer’s disease (AD) even in the absence of symptoms. As we better understand the role of biomarker accumulation in the clinical manifestation of AD, disclosing personal biomarker information will become increasingly relevant. Yet, interest and preferences for AD biomarker disclosure are not well understood. We developed a 30-minute phone survey to gather information from Black and white participants on likelihood to enroll in biomarker disclosure studies, reasons for enrolling, and potential outcomes following a hypothetical positive result. Data were collected from cognitively healthy participants (n=334, mean age=64.8±7.7, 45% Black) enrolled in the Wisconsin Alzheimer’s Disease Research Center or Wisconsin Registry for Alzheimer’s Prevention. 49.7% of participants were very or extremely likely to enroll in an AD biomarker disclosure study. This result varied by biomarker method, with about half the sample very or extremely likely to enroll in PET scan disclosure (45.5%), fewer likely to enroll in cerebrospinal fluid disclosure (32.2%), and a majority likely to enroll in blood-based biomarker disclosure (86.2%). The most important reasons for learning biomarker results included informing lifestyle changes to help prevent dementia (82.9% responded very or extremely important) and knowing more about personal AD risk (69.1% responded very or extremely important). These results suggest that as biomarker collection method burden decreases, willingness to participate in a biomarker disclosure study increases. Further, personal dementia prevention and risk are a strong motivator for learning biomarker results. Moving forward, these results may inform AD biomarker protocol development.

